# Improvement in Plasma Drug Activity during the Early Treatment Interval among Tanzanian Patients with Multidrug-Resistant Tuberculosis

**DOI:** 10.1371/journal.pone.0122769

**Published:** 2015-03-27

**Authors:** Norah D. Ndusilo, Scott K. Heysell, Stellah G. Mpagama, Jean Gratz, Farida H. Segesela, Saumu J. Pazia, Xin-Qun Wang, Eric R. Houpt, Gibson S. Kibiki

**Affiliations:** 1 Kilimanjaro Clinical Research Institute, Moshi, Tanzania; 2 Division of Infectious Diseases and International Health, University of Virginia, Charlottesville, United States of America; 3 Kibong’oto Infectious Diseases Hospital, Sanya Juu, Tanzania; 4 Department of Public Health Sciences, University of Virginia, Charlottesville, United States of America; Hospital San Agustín. Aviles. Asturias. Spain, SPAIN

## Abstract

**Background:**

Individual pharmacokinetic variability may be common in patients treated for multidrug-resistant tuberculosis (MDR-TB) but data are sparse from resource-limited settings and across the early treatment interval.

**Methods:**

Plasma drug activity, as measured by the TB Drug Activity (TDA) assay at 2 and 4 weeks of treatment with a standardized MDR-TB regimen was performed in patients with pulmonary MDR-TB from Tanzania. TDA values were correlated with measures of early treatment outcome including every two week collection of sputum for time-to-positivity (TTP) in liquid culture from the MGIT 960 automated system. Patients were evaluated at 24 weeks and those surviving without delayed sputum culture conversion (>8 weeks), culture reversion after previously negative, or weight loss were defined as having a favorable outcome.

**Results:**

Twenty-five patients were enrolled with a mean age of 37 ±12 years. All were culture positive from the pretreatment sputum sample with a mean TTP in MGIT of 257 ±134 hours, and the median time to culture conversion on treatment was 6 weeks. Twenty patients (80%) had an increase in TDA, with the overall mean TDA at 2 weeks of 2.1 ±0.7 compared to 2.4 ±0.8 at 4 weeks (p = 0.005). At 2 weeks 13 subjects (52%) had a TDA value > 2-log killing against their own *M*. *tuberculosis* isolate compared to 17 subjects (68%) at 4 weeks (McNemar’s exact test p = 0.29). An interim treatment outcome was able to be determined in 23 patients (92%), of whom 7 had a poor outcome (30%). An increase in TDA from week 2 to week 4 was associated with favorable outcome, [unadjusted OR = 20.0, 95% CI: 1.61–247.98, exact p = 0.017 and adjusted OR = 19.33, 95% CI: 1.55–241.5, exact p = 0.023].

**Conclusions:**

The majority of patients with MDR-TB in Tanzania had an increase in plasma drug activity from week 2 to week 4 of treatment as measured by the TDA assay. Understanding the etiology and full impact of this dynamic may inform therapeutic intervention.

## Introduction

Multidrug-resistant tuberculosis (MDR-TB) treatment outcomes remain poor in part because second-line drugs are less potent and often necessitate prolonged treatment duration [1]. Acquired drug-resistance while on MDR-TB therapy is increasingly recognized [2]. We have previously demonstrated that pharmacokinetic variability is common for key drugs in a standard MDR regimen when examining drug concentrations after two weeks of treatment at the time of estimated peak (C_max_) [3]. However, little is known about the change in pharmacokinetics at different time points in the treatment interval, and whether such changes predict treatment outcome.

*In vivo* drug exposure can be approximated by use of *in vitro* models, for instance those that examine variable drug concentration relative to *Mycobacterium tuberculosis* growth [4]. One such test, the plasma TB Drug Activity (TDA) assay, is based on the original Schlichter bactericidal assay for endocarditis and similar whole-blood culture techniques for *M*. *tuberculosis* [5,6]. The TDA assay uses a patient’s own plasma collected during TB treatment at the time of estimated C_max_ and the patient’s own *M*. *tuberculosis* isolate, measuring the ratio of time-to-positivity of growth in the Bactec MGIT system (Becton Dickinson, USA) of the plasma co-cultured isolate to that without prior plasma co-culture [7]. The use of plasma without leukocytes constrains analysis to drug effect alone, and the standard pH of MGIT media negates the activity of pyrazinamide. Typically static drugs, such as ethambutol, have little activity in the assay, but in a MDR-TB regimen, the fluoroquinolone and aminoglycoside drugs are most active [7]. For example, in patients with MDR-TB in Tanzania, those with the lowest TDA value (<1.5) after 2 weeks of treatment, which was indicative of very poor *in vitro* killing, had lower levofloxacin and kanamycin C_2hr_/minimum inhibitory concentration (MIC) ratios than subjects with a TDA≥1.5 [3].

As high performance liquid chromatography for drug concentration monitoring is unavailable in our setting and most other TB endemic countries, we sought to compare plasma drug activity at 2 and 4 weeks of treatment with a standard MDR-TB regimen in patients with pulmonary MDR-TB from Tanzania and correlate activity with more precise measures of early treatment outcome.

## Materials and Methods

Patients referred for treatment of MDR-TB treatment at the Kibong’oto Infectious Diseases Hospital (KIDH) in Northern Tanzania were recruited for enrollment. Adult patients were included if initial screening of sputum by Xpert MTB/RIF assay (Cepheid, USA) was positive for *M*. *tuberculosis* complex and rifampin resistance. All subjects signed written informed consent and the study was approved jointly by the human subjects review boards: the Research Ethical Clearance Board at Tumaini University Makumira, the National Institute for Medical Research in Dar es Salaam Tanzania, and the Institutional Review Board for Human Subjects Research at the University of Virginia. All patients were initiated on a standardized intensive phase MDR-TB regimen per hospital protocol (kanamycin or capreomycin, levofloxacin, pyrazinamide, ethionamide, cycloserine and ethambutol if known susceptible) [8].

### Sputum processing and monitoring of clinical response

All research laboratory procedures were performed at the Kilimanjaro Clinical Research Institute (KCRI) Biotechnology Laboratory. Overnight pooled sputum was additionally collected for this study prior to MDR-TB treatment initiation [9], the ‘pretreatment’ specimen, and processed for culture in the Bactec MGIT 960 automated liquid media system (Becton Dickinson, USA) and on Lowenstein-Jensen media. Specimens were digested, decontaminated and concentrated using the N-acetylcysteine and sodium hydroxide methodology prior to equal inoculum distribution to the solid and liquid media. Cultures were incubated for a maximum of 6 weeks, flagged as positive by the hourly time-to-positivity (TTP) in the automated MGIT system, and inspected weekly for the solid media specimens. All cultured growth was confirmed as *M*. *tuberculosis* complex by DNA probe (Gen-Probe, USA).

Subjects were excluded if pretreatment sputum specimens were culture negative. Subsequent overnight pooled specimens were collected at 2, 4, 6, 8, 10 and 12 weeks after MDR-TB treatment initiation for culture in the MGIT system. Previously, sequential TTP in MGIT has demonstrated a consistent inverse correlation with counting of colony forming units to CFU on solid agar [10,11]. Additional clinical response was monitored per hospital protocol and included monthly sputum collection for mycobacterial culture on solid agar (Lowenstein-Jensen media) for a minimum of six months, documentation of death from any cause, inpatient treatment completion, and change in weight while on treatment.

### Plasma TB drug activity (TDA) determination

Blood was collected at week 2 and week 4 following MDR-TB treatment initiation. All daily medication was administered directly observed by research staff and 2 hours after administration, at the time of estimated peak plasma concentration (C_2hr_), venous blood was collected and centrifuged onsite. Plasma was transported on ice to the KCRI Biotechnology laboratory and stored at -80°C. TDA analyses of both week 2 and week 4 samples were performed simultaneously in batch, and all tests were in duplicate.

The TDA assay was performed as previously described [3]. Briefly, a 500 μl suspension of a 1:10 dilution of 0.5 McFarland of the subject’s pretreatment *M*. *tuberculosis* isolate was added to two 2 ml screw-top tubes for plasma co-culture, and the same 500 μl inoculum was added to two 7 ml MGIT tubes with 800 μl of Middlebrook 7H9+10%OADC which were incubated until TTP. The 2 ml screw-cap tubes were centrifuged, the supernatant discarded, and the pellet re-suspended in phosphate-buffered saline before 300 μl of plasma was added. This process was equivalent for both week 2 and week 4 plasma. Following 72 hours incubation, the plasma co-cultured tubes were centrifuged, the supernatant discarded, the pellet resuspended in sterile-distilled water. After vortexing and centrifugation, the supernatant was discarded and replaced with 500 μl of Middlebrook 7H9+10%OADC, and the mixture transferred to a new MGIT tube for incubation until TTP [3]. As previously described, a second set of control tubes were prepared for each isolate by further diluting the suspension 1:100 and adding 500 μl to MGIT tubes. TDA was reported as the ratio of TTP of plasma co-cultured TB in hours to the TTP of the control (identical 1:10 suspension), whereby a TDA of <1.0 represented growth, a TDA of 1.0 represented stasis, and a TDA of >1.0 represented killing. The ratio of the TTP of the 1:1,000 suspension to the 1:10 suspension was recorded to establish the TDA ratio equivalent to 2-log killing for each subject’s isolate.

### Statistical analysis

Basic demographic data and clinical information were abstracted from charts. Statistical analysis was performed using SAS 9.4 (Cary, U.S.A.). Changes in the mean TDA values between week 2 and 4 were determined by paired t-tests, and the difference in proportion with TDA value >2-log killing against their own *M*. *tuberculosis* isolate between week 2 and 4 was assessed by McNemar’s exact test. The linear relationship was analyzed by ANOVA for the continuous variables of TDA, the week of sputum culture conversion to negative in MGIT, and the change in TTP from the pretreatment to the week 4 specimen. For the change in TTP calculation, a culture negative week 4 specimen was imputed as TTP of 1008 hours (as cultures are incubated 42 days in MGIT system before negativity declared).

Additionally, a composite interim treatment outcome was determined by classifying patients as having a ‘poor’ outcome if the sputum culture was converted late (>8 weeks), the patient had culture reversion to positive after the week 12 sputum collection (determined by monthly sputum culture on Lowenstein-Jensen media between 12 and 24 weeks per hospital protocol), death after the week 4 blood collection, and/or the patient had lost weight at 24 weeks compared to the pretreatment weight. Patients surviving without late conversion, reversion or weight loss were classified as having a ‘favorable’ outcome. These variables were chosen for their clinical and programmatic significance [12], any one of which in our setting often leads to a MDR-TB patient remaining hospitalized for more intense monitoring and support. Due to the limited sample size, effects of each predictor of the interim treatment outcome were assessed using univariate exact logistic regression models [13]. For each of the significant predictors from the univariate analyses, we also performed multivariable exact logistic regression analyses by adjusting for pre-treatment TTP < 216 hours which previously has been associated with more extensive pulmonary disease by chest imaging and more prolonged infectiousness [14].

## Results

Twenty-seven patients were initially enrolled but two died prior to the blood draw for the second plasma sample and were therefore excluded from analysis. Hence, 25 subjects had both a week 2 and week 4 plasma collected for TDA. The mean age was 37 ±12 years and the majority were male (Table 1). Baseline demographics were similar to prior referral patterns to KIDH for MDR-TB treatment [8]. Twenty-two patients (88%) had prior TB treatment and while 7 were HIV infected, nearly all were on antiretroviral therapy at the time of MDR-TB treatment initiation. Only 3 patients received capreomycin, all others received kanamycin.

**Table 1:** Clinical characteristics, patients treated for pulmonary multidrug-resistant tuberculosis (MDR-TB), N = 25.

Demographic	Result
Age, mean years ±SD	37 ±12
Gender, male (%)	17 (68)
Body Mass Index^a^, mean % ±SD	19.9 ±3.4
HIV infected	7 (28)
Mean CD4 count^a^, cells mm^3^ ±SD	247 ±111
On antiretroviral therapy^a^ (% HIV infected)	6 (86)
Smoking	7 (28)
Alcohol use	10 (40)
Prior TB treatment episodes	
None	3 (12)
One	7 (28)
Two or more	15 (60)

^a^At MDR-TB treatment initiation.

### Dynamics of sputum culture conversion

**Fig 1:**
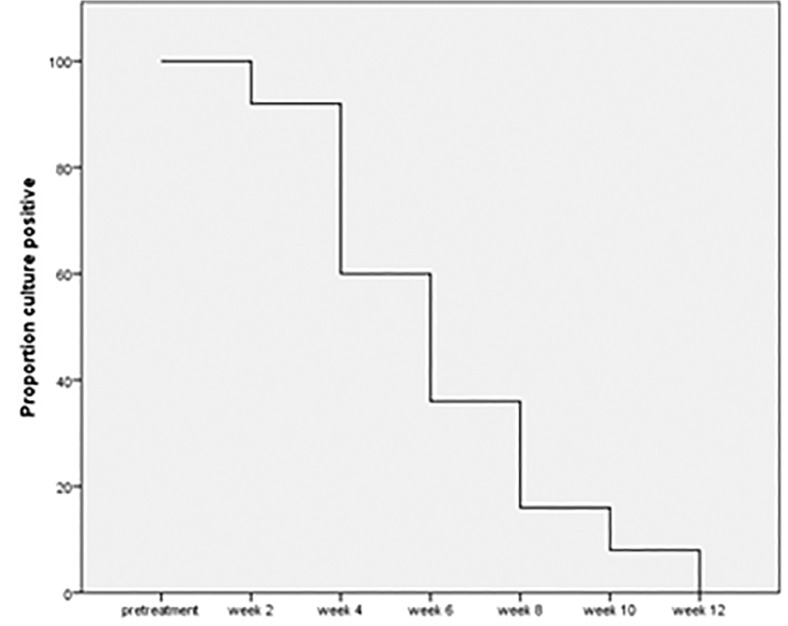
Sputum culture positive by week in MGIT system, N = 25. All patients (100%) culture positive from pretreatment sputum specimen.

All patients were culture positive from the pretreatment sputum sample with a mean TTP in MGIT of 257 ±134 hours (minimum 123 hours; maximum 588 hours). Eleven patients (44%) had a TTP of less than 216 hours (9 days). The median time to culture conversion was 6 weeks (minimum 2 weeks; maximum 12 weeks). Twenty-one patients (84%) were culture negative at 8 weeks (Fig. 1). Two patients that were culture negative at 2 weeks, were again culture positive at 10 weeks (though of low bacterial burden, TTP in MGIT of 725 and 935 hours at 10 weeks), but ultimately culture negative again at 12 weeks and upon subsequent monthly evaluations per hospital protocol. Thus, these patients were classified as culture converted at 12 weeks.

### Dynamics of plasma drug activity

**Fig 2:**
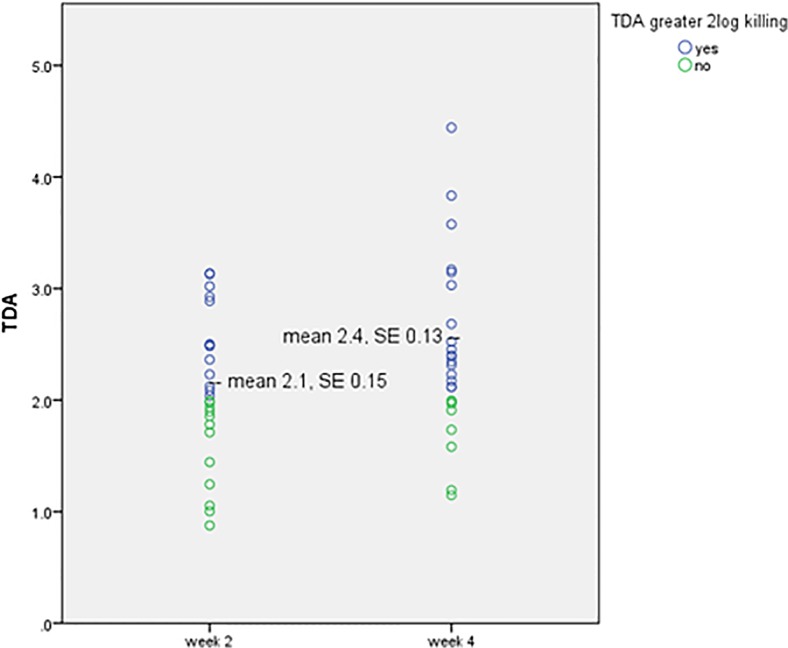
Change in plasma drug activity measured at the time of estimated peak concentration (2hr) after 2 and 4 weeks of treatment. TDA = Tuberculosis drug activity, where values greater than 1.0 indicate *in vitro* killing of a patient’s plasma against their own *M*. *tuberculosis* isolate. p = 0.005 by paired sample t-test.

Differences were noted between the week 2 and week 4 plasma drug activity. Twenty patients (80%) had an increase in TDA values, with the overall mean TDA at 2 weeks of 2.1 ±0.7 compared to 2.4 ±0.8 at 4 weeks (p = 0.005) (Fig. 2). At 2 weeks 13 subjects (52%) had a TDA value > 2-log killing against their own *M*. *tuberculosis* isolate compared to 17 subjects (68%) at 4 weeks (McNemar’s exact test: p = 0.29).

While the week of culture conversion did not correlate with the week 2 TDA (R^2^ = 0.04, p = 0.35), week 4 TDA (R^2^ = 0.07, p = 0.2), or the cumulative weeks 2 and 4 values (R^2^ = 0.06, p = 0.22), a linear correlation was found with the change in TTP from the pretreatment specimen to the week 4 specimen and both the week 2 TDA (R^2^ = 0.24, p = 0.06) and the week 4 TDA (R^2^ = 0.27, p = 0.04). In addition, higher TDA values on average were found in patients that converted by 8 weeks compared to those with culture conversion >8 weeks (mean two week TDA 2.1 ±0.69 in early converters v. 1.89 ±0.38 in late converters, p = 0.37; mean four week TDA 2.5 ±0.78 v. 2.0 ±0.61, p = 0.21), yet these differences did not approach statistical significance given the small sample of patients with conversion >8 weeks.

### Interim treatment outcome

One patient was transferred out of care and another defaulted treatment, allowing the interim outcome to be determined in 23 patients (92%), of whom 7 had a poor outcome (30%) including 1 with culture reversion and 3 others with weight loss (Table 2 and S1 Table). Unexpectedly, all HIV infected patients had a favorable outcome. In univariate exact logistic regression analysis, the total TDA value at 4 weeks (exact p = 0.033), a TDA value > 2-log killing at 4 weeks (OR = 10.83, 95% CI: 1.37–85.43, exact p = 0.026), and an increase in TDA from week 2 to week 4 (OR = 20.0, 95% CI: 1.61–247.98, exact p = 0.017) appeared protective for poor outcome. After adjusting for pre-treatment TTP < 216 hours, either a TDA value > 2-log killing at 4 weeks (OR = 16.84, 95% CI: 1.46–194.58, exact p = 0.02) or an increase in TDA from week 2 to week 4 (OR = 19.33, 95% CI: 1.55–241.51, exact p = 0.023) remained statistical significant, and the total TDA value at 4 weeks was marginally significant (exact p = 0.055). The TDA increase from Week 2 to week 4 corresponded to a high probability of the favorable outcome (predicted probability: 0.77–0.82) (S2 Table).

**Table 2:** Predictors of favorable interim treatment outcome using exact logistic regression.

Characteristic	Favorable N = 16	PoorN = 7	Unadjusted odds ratio [95% CI]	Exact p-value
Age, years				
<30	2 (12)	2 (29)	referent	
30–49	11 (69)	4 (57)	2.75 [0.28–26.6]	p = 0.56
≥50	3 (19)	1 (14)	3.0 [0.15–59.9]	p = 0.99
Gender, male	10 (63)	6 (86)	0.28 [0.03–2.9]	p = 0.37
Baseline Body Mass Index, mean % ±SD	19.4 ±2.0	19.6 ±4.6		p = 0.68
HIV infected	6 (38)	0	n/c	p = 0.12
Smoking	3 (19)	4 (57)	0.17 [0.03–1.22]	p = 0.14
Alcohol	6 (38)	4 (57)	0.45 [0.45–2.74]	p = 0.65
Prior TB treatment episodes				
None	3 (19)	0	referent	
One	3 (19)	2 (29)	n/c	P = 0.46
Two or more	10 (62)	5 (71)	n/c	P = 0.52
Pretreatment MGIT TTP^a^, mean hours ±SD	261 ±155	244 ±108		p = 0.93
Proportion with pretreatment MGIT TTP <216 hours	9 (56)	3 (43)	1.71 [0.29–10.3]	p = 0.67
Pretreatment to week 4 change in MGIT TTP^a^, mean hours ±SD	562 ±360	433 ±324		p = 0.46
Week 2 TDA, mean ±SD	2.2 ±0.67	1.9 ±0.54		p = 0.38
Proportion with week 2 TDA > 2log killing, (%N)	9 (56)	3 (43)	1.71 [0.29–10.3]	p = 0.67
Week 4 TDA mean	2.6 ±0.75	1.9 ±0.53		p = 0.033
Proportion with week 4 TDA > 2log killing, (%N)	13 (81)	2 (28)	1.71 [0.29–10.3]	p = 0.026
Proportion with increase in TDA from week 2 to week 4	15 (94)	3 (43)	20.0 [1.61–247.98]	p = 0.017

^a^Greater change in time-to-positivity (TTP) reflects greater decrement in sputum bacterial burden.

## Discussion

Patients with MDR-TB in Tanzania experienced an increase in plasma drug activity from week 2 to week 4 of treatment with a standardized regimen as measured by the TDA assay. Despite the majority of patients achieving early sputum culture conversion, the subjects’ plasma drug activity was associated with a greater decrement in the sputum bacterial burden from the pretreatment to the week 4 sample. Furthermore, an increase in plasma drug activity from week 2 to week 4 appeared to associate with a favorable interim treatment outcome. These findings are important because they highlight a dynamic pattern of drug exposure in individual patients and emphasize that pharmacokinetic or bactericidal studies in MDR-TB should be performed a multiple time-points within the treatment interval.

The median time to sputum culture conversion was relatively rapid compared to other MDR-TB cohorts [15] and occurred as early as two weeks in some patients, but may also reflect the increased frequency of sampling (every two weeks compared to monthly in many settings). Nevertheless these findings remain consistent with the first cohort of patients treated for MDR-TB at Kibong’oto where 89% successfully completed the intensive phase of therapy and were discharged from the hospital with a median time to culture conversion of 2 months [8]. Considerable delay still exists from the time of diagnosis to MDR-TB treatment in Tanzania, which may have selected for subjects relatively healthy enough to survive to treatment initiation. Similarly, 6 of 7 HIV patients were already on antiretroviral treatment at the time of presentation as otherwise poor outcome would have been hypothesized to be more common in this subgroup.

While the quantitative bacterial burden in pretreatment sputum as measured by TTP in MGIT has been associated with increased transmission and extent of disease on chest imaging [14], we did not find a correlation with pretreatment TTP and week of culture conversion nor did we observe differences in pretreatment TTP among patients with favorable or poor interim treatment outcome. A greater influence of pretreatment bacterial burden may have been observed with a larger patient sample size.

Of additional relevance, acquired drug-resistance on MDR-TB treatment has been documented to occur in as many as 16% of cases in some settings despite adherence to WHO/Green Light Committee standards and oversight [2], raising concern that individual pharmacokinetic variability may be playing a role. In a recent large study of drug-susceptible TB, suboptimal pharmacokinetics to isoniazid and/or rifampin was present in all cases of acquired drug-resistance to these medications [16]. One patient was documented to have late relapse in our study, but repeat second-line drug susceptibility was not performed. Nevertheless, our findings suggest that prospective studies of MDR-TB pharmacokinetics would be beneficial to determine if threshold drug concentrations for key drugs would ultimately predict which patients would be at most risk for the longer term outcome of relapse with or without acquired drug-resistance.

A limitation of the study is that the assay of plasma drug activity is crude, and while predominately measuring the killing of the fluoroquinolone and aminoglycoside in a standard MDR-TB regimen [3], it does not provide the precision of concurrently measuring individual drug concentrations and performing quantitative susceptibility on the *M*. *tuberculosis* isolate. Therefore, it is unclear if a particular drug is primarily responsible for the observed improvement in week 2 to week 4 plasma drug activity values. Also, blood was collected only at 2 hours after medication administration at the time of estimated C_max_ and thus theoretically the improvement in drug activity may have represented a shift in the time to C_max_ rather than a true peak increase. Yet provocative pharmacokinetic study from Peru has suggested that TB patients had a lower intestinal area of absorption compared to healthy controls which was even more pronounced for MDR-TB [17]. It is possible then during treatment for MDR-TB and concomitant nutritional support per hospital protocol that intestinal absorption of medications was improved. While this may be most applicable to the fluoroquinolone [18], the hypothesis is supported by the trend of lack of improvement in plasma drug activity with poor interim treatment outcome which included lack of weight gain. Of the 3 patients with documented lack of weight gain, 2 (67%) did not have a week 4 TDA value that exceeded a 2log killing. Further pharmacokinetic testing in conjunction with newly validated serum and fecal markers of intestinal absorption and enteropathy may be additionally revealing [19].

## Conclusions

In summary, individual plasma drug activity appears dynamic throughout the early treatment interval of MDR-TB. Future pharmacokinetic studies should account for this expectation, while understanding the etiology and full impact of this dynamic may inform therapeutic intervention.

## Supporting Information

S1 Table Parameter estimates of the interim treatment outcome in the exact logistic regression model. (PDF)

S2 Table Predicted probabilities of favorable treatment outcome. Note: Supplemental Table 2 demonstrates that TDA increase from Week 2 to week 4 corresponded to high predicted probabilities of the favorable outcome
(PDF)
