# Supplementary material for: Improvement in Plasma Drug Activity during the Early Treatment Interval among Tanzanian Patients with Multidrug-Resistant Tuberculosis
Source: PLoS One. 2015 Mar 27;10(3):e0122769. doi: 10.1371/journal.pone.0122769 (PMC4376785; doi:10.1371/journal.pone.0122769)
Supplement: S1 Table — (PDF) [file pone.0122769.s001.pdf]

S1 Table. **Parameter estimates of the interim treatment outcome in the exact logistic regression model**

**Variable**	**Alpha (±standard error)**	**Beta (±standard error)**
Age, years		
<30	referent	
30-49	0.51±0.58	0.51±0.58
≥50	0.55±0.76	0.55±0.76
Gender, male	1.79±1.08	-1.28±1.20
Baseline Body Mass Index, mean % ±SD	1.37±3.09	-0.03±0.16
HIV infected, yes	0.36±0.49	12.07±203.40
Smoking	1.47±0.64	-1.75±1.00
Alcohol	1.20±0.66	-0.80±0.92
Prior TB treatment episodes		
None	referent	-5.58±93.75
One	5.99±93.75	-5.90±148.50
Two or more	6.59±148.50	
Pretreatment MGIT TTP*	0.60±0.96	0.001±0.003
Proportion with pretreatment MGIT TTP <216 hours	0.56±0.63	0.54±0.92
Pretreatment to week 4 change in MGIT TTP*	0.21±0.80	0.001±0.001
Week 2 TDA	-0.66±1.59	0.73±0.77
Proportion with week 2 TDA > 2log killing, (%N)	0.56±0.63	0.54±0.92

Week 4 TDA mean	-3.84±2.46	2.14±1.16
Proportion with week 4 TDA > 2log killing, (%N)	-0.51±0.73	2.38±1.05
Proportion with increase in TDA from week 2 to week 4	-1.39 ±1.12	3.00 ±1.28
