# Supplementary material for: Improvement in Plasma Drug Activity during the Early Treatment Interval among Tanzanian Patients with Multidrug-Resistant Tuberculosis
Source: PLoS One. 2015 Mar 27;10(3):e0122769. doi: 10.1371/journal.pone.0122769 (PMC4376785; doi:10.1371/journal.pone.0122769)
Supplement: S2 Table — Note: Supplemental Table 2 demonstrates that TDA increase from Week 2 to week 4 corresponded to high predicted probabilities of the favorable outcome (PDF) [file pone.0122769.s002.pdf]

S2 Table. Predicted probabilities of favorable treatment outcome

**TDA increase from Week 2 to week 4**	**Pretreatment TTP < 216 hours**	**Predicted probabilities of favorable outcome**
**Yes**	**Yes**	**0.8217**
No	Yes	0.2550
No	No	0.2031
**Yes**	**No**	**0.7743**

Note: Supplemental Table 2 demonstrates that TDA increase from Week 2 to week 4 corresponded to high predicted probabilities of the favorable outcome.
